# Supplementary material for: Asthma reduces glioma formation by T cell decorin-mediated inhibition of microglia
Source: Nat Commun. 2021 Dec 8;12:7122. doi: 10.1038/s41467-021-27455-6 (PMC8654836; doi:10.1038/s41467-021-27455-6)
Supplement: Supplementary file 1 — Supplementary Information [file 41467_2021_27455_MOESM1_ESM.pdf]

Supplementary Information

Asthma reduces glioma formation by T cell decorin-mediated inhibition of microglia

Jit Chatterjee, Shilpa Sanapala, Olivia Cobb, Alice Bewley, Andrea K. Goldstein, Elizabeth Cordell, Xia Ge, Joel R. Garbow, Michael J. Holtzman, David H. Gutmann

Departments of [1]Neurology,[2] Mallinckrodt Institute of Radiology and [3]Pulmonary and Critical Care Medicine, Washington University School of Medicine, St. Louis MO

Address correspondence to: David H. Gutmann, MD, PhD, Department of Neurology, Washington University, Box 8111, 660 S. Euclid Avenue, St. Louis, MO, USA 63110. 314-362-7379 (phone); gutmannd@wustl.edu (email); ORCID #0000-0002-3127-5045

Supplementary Figures

Supplementary Fig 1

Supplementary Fig 2

Supplementary Fig 3

Supplementary Fig 4

Supplementary Fig 5

Supplementary Fig 6

Supplementary Tables

Supplementary Table 1

Supplementary Table 2

a

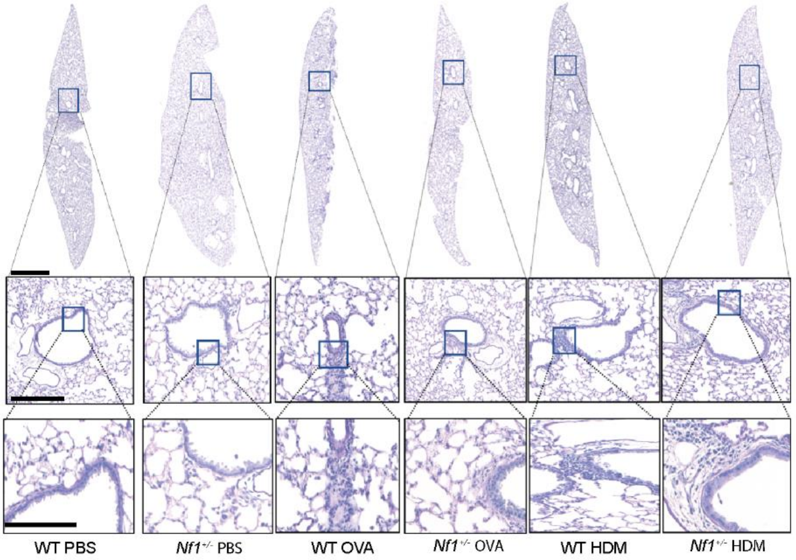
WT PBS
Nf1+/- PBS
WT OVA
Nf1+/- OVA
WT HDM
Nf1+/- HDM

b

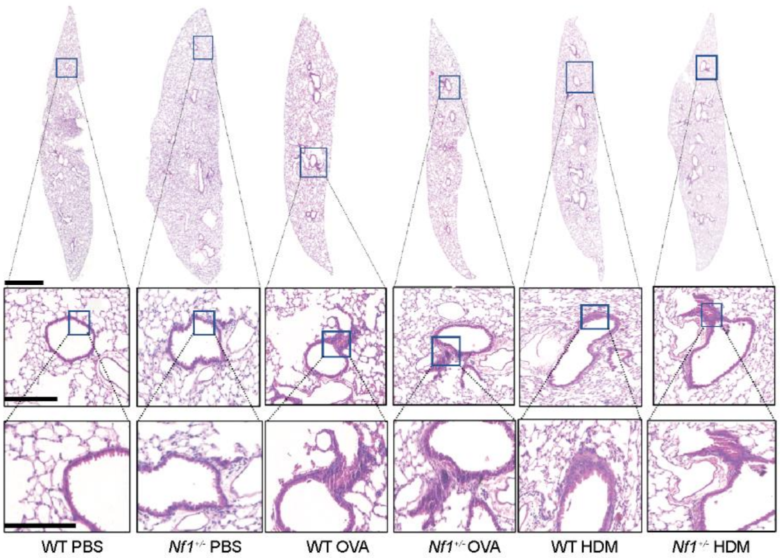
WT PBS
Nf1+/- PBS
WT OVA
Nf1+/- OVA
WT HDM
Nf1+/- HDM

c

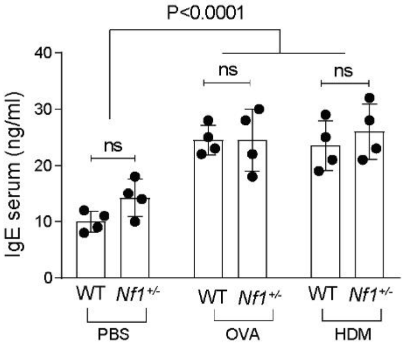
P<0.0001
ns
ns
ns
IgE serum (ng/ml)
40
30
20
10
0
WT
Nf1+/-
WT
Nf1+/-
WT
Nf1+/-
PBS
OVA
HDM

Supplementary Fig. 1. WT and *Nf1*$^{+/-}$ mice were treated between 4-6 weeks of age with either OVA or HDM (experimental asthma) or PBS (controls). Representative **(a)** H&E and **(b)** PAS-stained images of the lungs from WT and *Nf1*$^{+/-}$ mice treated with PBS (*n*=6), HDM (*n*=6) and OVA (*n*=6). Increased airway wall thickening and immune cell infiltration into bronchial and peri-bronchial sites were observed 7 days after OVA and HDM treatment. Boxes indicate thickened bronchial walls. Small scale bar, 500 mm; large scale bar, 250 mm. **(c)** Similar increases in serum IgE were observed in WT (*n*=4) and *Nf1*$^{+/-}$ (*n*=4) mice treated with OVA and HDM relative to PBS-treated control mice. Data are presented as the mean ± SEM. One-way ANOVA with Bonferroni post-test correction; n.s., not significant; Exact P values are indicated within each panel. From left to right in each panel: (**c**) ns, ns, ns, P<0.0001.

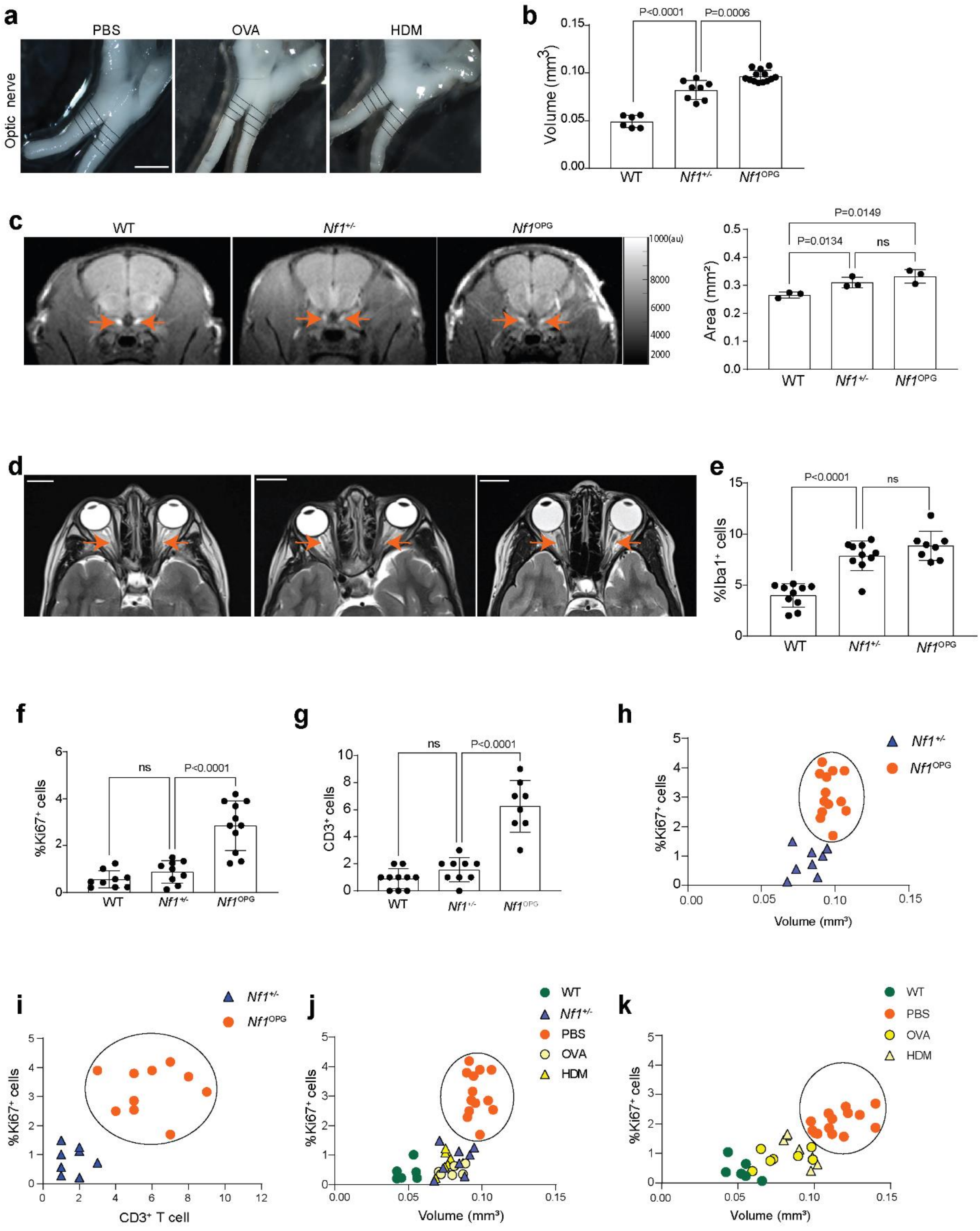

a
PBS
OVA
HDM
Optic nerve
b
Volume (mm3)
P<0.0001
P=0.0006
WT
Nf1+/-
Nf1OPG
c
WT
Nf1+/-
Nf1OPG
1000(au)
8000
6000
4000
2000
Area (mm2)
P=0.0149
P=0.0134
ns
d
e
%Iba1+ cells
P<0.0001
ns
f
%Ki67+ cells
ns
P<0.0001
g
CD3+ cells
ns
P<0.0001
h
%Ki67+ cells
Volume (mm3)
i
%Ki67+ cells
CD3+ T cell
j
WT
Nf1+/-
PBS
OVA
HDM
%Ki67+ cells
Volume (mm3)
k
WT
PBS
OVA
HDM
%Ki67+ cells
Volume (mm3)

Supplementary Fig. 2. (a) Representative images of murine optic nerves from PBS-, OVA-, and HDM-treated *Nf1*^{OPG} mice. The black lines represent the diameters used to generate the optic nerve volumes. **(b)** Optic nerve volumes from *Nf1*$^{+/-}$ (n=8) and *Nf1*^{OPG} mice (n=13) compared to WT controls (n=6). Bar graphs represent the means ± SEM of independent biological samples. **(c)** Thickened optic nerves were observed in *Nf1*$^{+/-}$ mice by $MnCl_2$-enhanced T1-weighted magnetic resonance imaging and area measurements. Bar graphs represent the means ± SEM of WT, *n* = 3, *Nf1*$^{+/-}$, *n* = 3, *Nf1*^{OPG}, *n* = 3, independent biological samples. Red arrows point to the optic nerves. **(d)** Thickened optic nerves were also seen on T2-weighted axial magnetic resonance images from representative individuals with NF1, but without an OPG (middle panel), relative to those with an OPG (right panel) or an individual without NF1 (left panel). Red arrows point to the optic nerves. **(e)** Microglia content (%Iba1^{+} cells) was increased in the optic nerves of *Nf1*$^{+/-}$ (*n*=10) and *Nf1*^{OPG} (*n*=8) mice relative to WT mice (*n*=10). Bar graphs represent the means ± SEM of independent biological samples. **(f)** Optic nerve proliferation (%Ki67^{+} cells) was increased in *Nf1*^{OPG} mice (*n*=11) compared to *Nf1*$^{+/-}$ (*n*=9) and WT mice (*n*=9). All data are presented as the mean ± SEM. **(g)** CD3^{+} T cell content was increased in the optic nerves from *Nf1*^{OPG} (*n*=8) mice relative to WT (*n*=10) and *Nf1*$^{+/-}$ (*n*=9) mice. All data are presented as the mean ± SEM. **(h)** Graph demonstrating that optic gliomas (increased %Ki67^{+} cells and volumes) form in *Nf1*^{OPG} (*n*=13), but not in *Nf1*$^{+/-}$ (*n*=8), mice. **(i)** Graph demonstrating that optic gliomas form in *Nf1*^{OPG} (*n*=10), but not in *Nf1*$^{+/-}$ (*n*=8), mice (increased %Ki67^{+} cells and CD3^{+} cell content). Graphs demonstrating that **(j)** OVA (*n*=8) and HDM (*n*=8) treatments prevent optic glioma formation (12 weeks of age), with %Ki67^{+} cells and volumes comparable to *Nf1*$^{+/-}$ mice (*n*=8). **(k)** Graph demonstrating durable effects of OVA (*n*=7) and HDM (*n*=8) treatments in *Nf1*^{OPG} mice at 6 months of age. Scale bars, **(a)** 100 µm. **(d)** Scale bars 2cm. One-way ANOVA with Bonferroni post-test correction; n.s., not significant; Exact P values are indicated within each panel. From left to right in each panel: (**b**) P<0.0001, P=0.0006; (**c**) P=0.0134, ns, P=0.0149; (**e**) P<0.0001, ns; (**f**) ns, P<0.0001; (**g**) ns, P<0.0001.

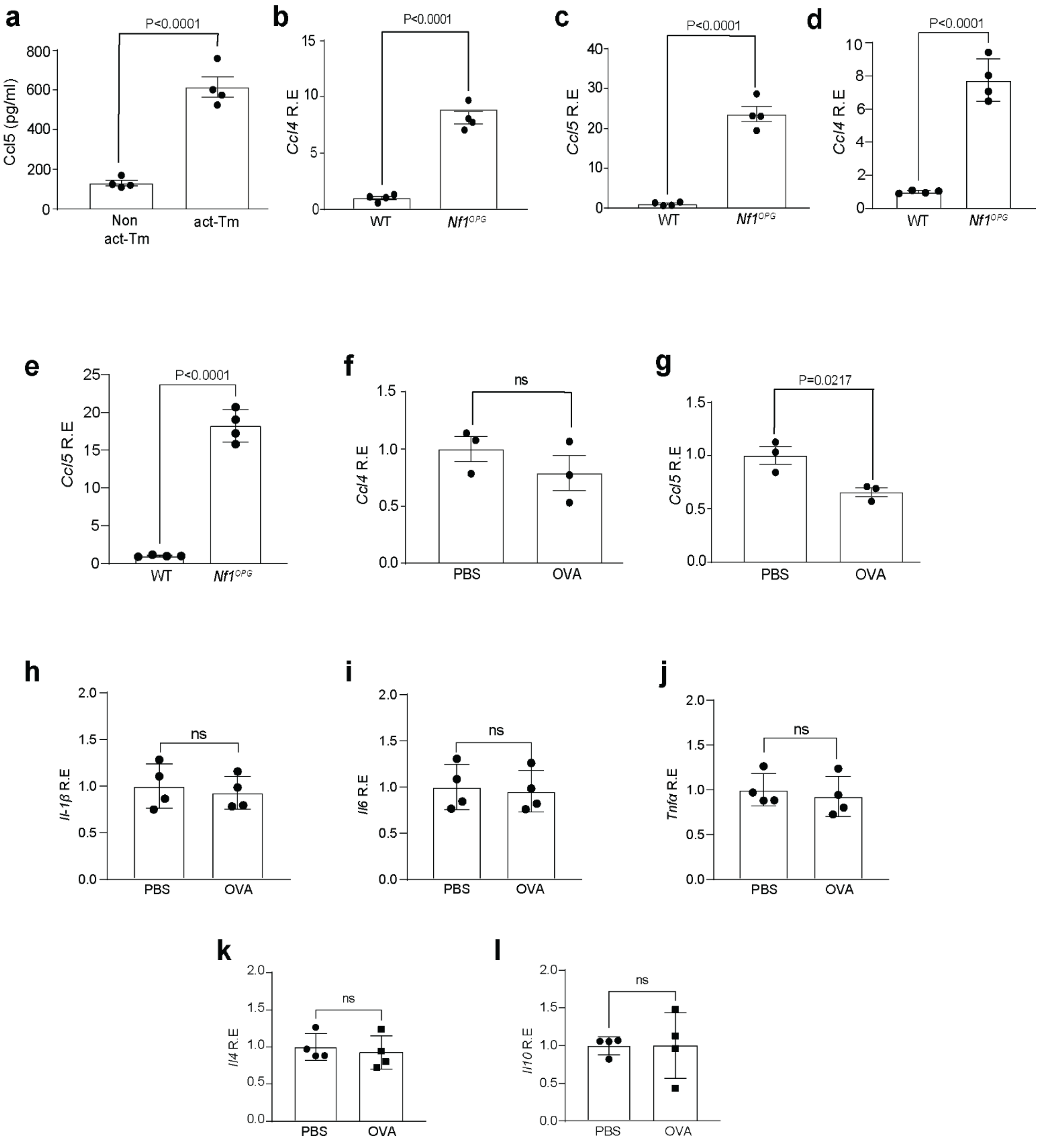

a
Ccl5 (pg/ml)
P<0.0001
800
600
400
200
0
Non act-Tm
act-Tm
b
Ccl4 R.E
P<0.0001
15
10
5
0
WT
Nf1OPG
c
Ccl5 R.E
P<0.0001
40
30
20
10
0
WT
Nf1OPG
d
Ccl4 R.E
P<0.0001
10
8
6
4
2
0
WT
Nf1OPG
e
Ccl5 R.E
P<0.0001
25
20
15
10
5
0
WT
Nf1OPG
f
Ccl4 R.E
ns
1.5
1.0
0.5
0.0
PBS
OVA
g
Ccl5 R.E
P=0.0217
1.5
1.0
0.5
0.0
PBS
OVA
h
Il-1β R.E
ns
2.0
1.5
1.0
0.5
0.0
PBS
OVA
i
Il6 R.E
ns
2.0
1.5
1.0
0.5
0.0
PBS
OVA
j
Tnfα R.E
ns
2.0
1.5
1.0
0.5
0.0
PBS
OVA
k
Il4 R.E
ns
2.0
1.5
1.0
0.5
0.0
PBS
OVA
l
Il10 R.E
ns
2.0
1.5
1.0
0.5
0.0
PBS
OVA

Supplementary Fig. 3. (**a**) Conditioned medium was collected from 2.5×10^6 cells ml^{-1} mouse splenic $CD3^+$ T cells seeded in complete RPMI 1640 medium following 2 days of CD3/CD28 stimulation (activated; act-Tm) or vehicle (PBS) treatment (non-activated; non-act-Tm). Higher Ccl5 levels were detected in act-Tm, relative to non-act-Tm, by ELISA (*n*=4). *Nf1*^{OPG} mouse optic nerves (*n*=4) have higher RNA expression of (**b**) *Ccl4* and (**c**) *Ccl5* at 12 weeks and 24 weeks (**d-e**) relative to WT mice. **(f)** No change in *Ccl4* RNA expression in the optic nerves from OVA-treated *Nf1*^{OPG} mice was observed relative to PBS-treated *Nf1*^{OPG} mice at 24 weeks of age by qRT-PCR (*n*=3). **(g)** *Ccl5* expression was reduced relative to PBS-treated *Nf1*^{OPG} mice by qRT-PCR (*n*=3). OVA- and PBS-treated *Nf1*^{OPG} mouse optic nerves (*n*=4) have similar expression of M1 markers [(**h**) *Il1β*, (**i**) Il6, (**j**) *Tnfα*] and M2 markers [(**k**) *Il4*, (**l**) *Il10*]. All data are presented as the mean ± SEM. A two-tailed Student's *t*-test was used. P values are indicated within each panel. From left to right in each panel: (**a**) P<0.0001; (**b**) P<0.0001; (**c**) P<0.0001; (**d**) P<0.0001; (**e**) P<0.0001; (**f**) ns; (**g**) P=0.0217; (**h**) ns; (**i**) ns; (**j**) ns; (**k**) ns; (**l**) ns.

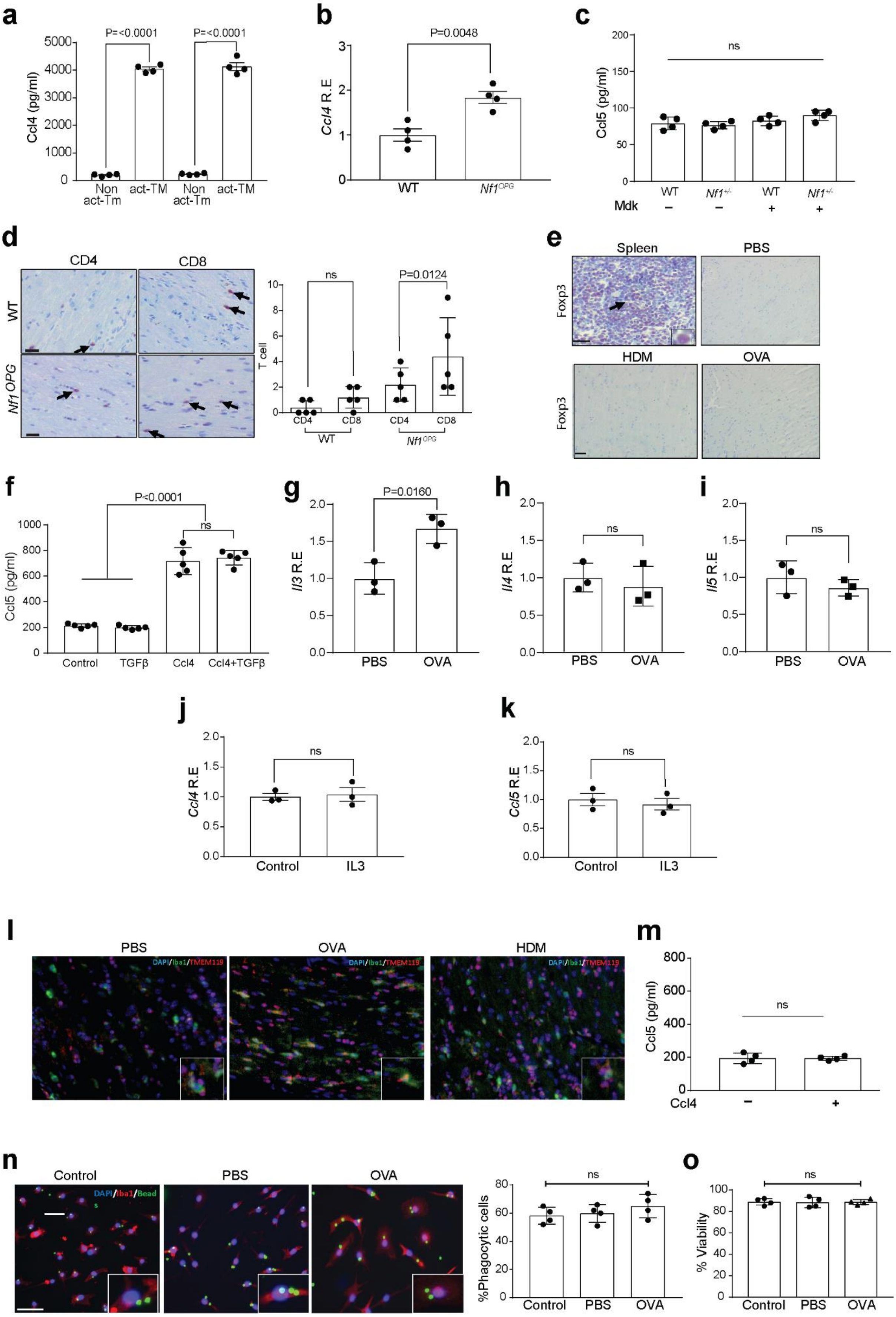

a
P=<0.0001
P=<0.0001
Ccl4 (pg/ml)
Non act-Tm
act-TM
Non act-Tm
act-TM
b
P=0.0048
Ccl4 R.E
WT
Nf1OPG
c
ns
Ccl5 (pg/ml)
WT
Nf1+/-
WT
Nf1+/-
Mdk
d
CD4
CD8
WT
Nf1 OPG
ns
P=0.0124
T cell
CD4
CD8
CD4
CD8
WT
Nf1OPG
e
Spleen
PBS
Foxp3
HDM
OVA
Foxp3
f
P<0.0001
ns
Ccl5 (pg/ml)
Control
TGFβ
Ccl4
Ccl4+TGFβ
g
P=0.0160
Il3 R.E
PBS
OVA
h
ns
Il4 R.E
PBS
OVA
i
ns
Il5 R.E
PBS
OVA
j
ns
Ccl4 R.E
Control
IL3
k
ns
Ccl5 R.E
Control
IL3
l
PBS
OVA
HDM
DAPI/Iba1/TMEM119
m
ns
Ccl5 (pg/ml)
Ccl4
n
Control
PBS
OVA
DAPI/Iba1/Beads
ns
%Phagocytic cells
Control
PBS
OVA
o
ns
% Viability
Control
PBS
OVA

Supplementary Fig. 4. (a) Conditioned medium was collected from 2.5×10^6 cells ml^{-1} PBS- and OVA treated mouse splenic $CD3^+$ T cells (*n*=4) seeded in complete RPMI 1640 medium following 2 days of CD3/CD28 stimulation (activated; act-Tm) or vehicle (PBS) treatment (non-activated; non-act-Tm). **(b)** Similar increases in Ccl4 were detected in the conditioned medium from PBS- and OVA-treated act-Tm by ELISA (*n*=4). A one-way ANOVA with Bonferroni post-test correction was used. (**c**) Midkine (100ηg/ml) treatment of WT and $Nf1^{+/-}$ microglia (*n*=4) for 48 h does not increase Ccl5 production. (**d**) Increased $CD8^+$ T cell content was observed in the optic nerves from $Nf1^{OPG}$ relative to WT mice. Similar $CD4^+$ T cell content was observed in $Nf1^{OPG}$ and WT optic nerves. Black arrows denote representative immunopositive cells (*n*=5). (**e**) No $Foxp3^+$ cells were detected in the optic nerves from 12-week-old $Nf1^{OPG}$ mice treated with PBS, OVA or HDM. (**f**) WT microglia were treated with either TGFβ (5ηg/ml) alone or in combination with Ccl4 (6000pg/ml). TGFβ does not induce microglia Ccl5 production, either alone or in combination with Ccl4 (*n*=5). One-way ANOVA with Bonferroni post-test correction was used. **(g-i)** *Il3*, *Il4* and *Il5* gene expression was examined in PBS- and OVA-treated $Nf1^{OPG}$ optic nerves (*n*=3), and only *Il3* expression was higher in OVA-treated $Nf1^{OPG}$ mice. A two-tailed Student's *t*-test was used. **(j-k)** WT T cell (*n*=3) and microglia (*n*=3) were treated with IL-3 (1 ng/mL). No change in T cell Ccl4 or microglia Ccl5 production was observed following treatment with IL-3. A two-tailed Student's *t*-test was used. (**l**) 12-week-old $Nf1^{OPG}$ optic nerves (*n*=5) following PBS, OVA, and HDM treatment were co-labelled with Iba1 and Tmem119 antibodies. Nearly all of the $Iba1^+$ cells were $Tmem119^+$ microglia, rather than Tmem119-negative macrophages. **(m)** Mouse splenic macrophages failed to induce Ccl5 expression in response to Ccl4. OVA treatment of mice does not change microglia **(n)** phagocytosis or **(o)** viability (n=4, 66.88±4.09% phagocytic cells). Scale bar, 25µm. A one-way ANOVA with Bonferroni post-test correction was used. Exact P values are indicated within each panel. N.S.; not significant. From left to right in each panel: (**a**) P<0.0001, P<0.0001; (**b**) P=0.0048; (**c**) ns, P=0.0124; **(d)** ns, P=0.0124; **(f)**) P<0.0001; (**g**) P=0.0160; (**h**) ns; (**i**) ns; (**j**) ns; (**k**) ns; (**m**) ns; **(n)** ns; **(o)** ns.

a

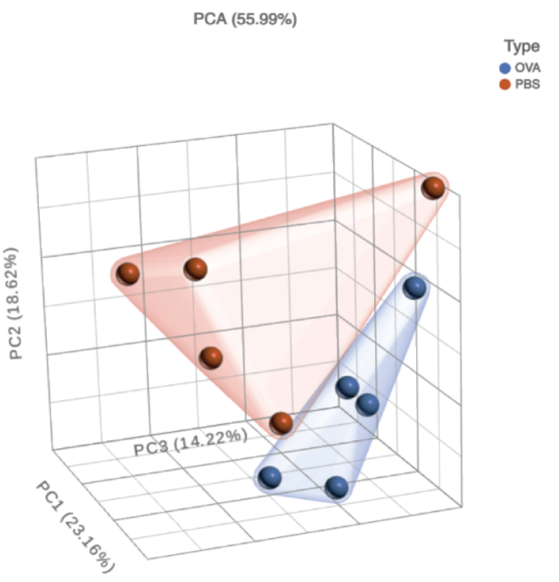

b

Ensembl	Total counts	P-value (OVA vs. PBS)	FDR step up (OVA vs. PBS)	Fold change (OVA vs. PBS)
Xist	1949.79	1.71E-08	1.63E-04	420.93
Tsix	963.15	3.30E-07	3.92E-04	96.47
Dcn	104.81	7.14E-08	1.97E-04	64.16
Gm43024	100.96	4.75E-06	2.97E-03	41.33
Stx19	53.26	7.76E-05	1.70E-02	31.20
Olfm5	32.82	1.23E-04	2.23E-02	30.72
Col6a5	32.56	1.54E-04	2.59E-02	30.40
Flrt2	31.18	4.25E-04	4.87E-02	28.89
Gm7265	40.35	2.77E-04	3.82E-02	24.39
Gm48673	59.63	7.49E-05	1.68E-02	24.35
Gm48062	49.59	1.68E-04	2.71E-02	19.74
Ltf	15180.05	1.62E-04	2.64E-02	19.35
Cr2	389.07	2.56E-04	3.60E-02	17.80
Ngp	43183.77	1.33E-04	2.34E-02	16.50
Krt19	73.29	2.21E-04	3.29E-02	13.52
Zfp987	144.95	5.13E-06	2.97E-03	13.45
Fcer2a	694.10	2.56E-04	3.60E-02	12.94
C130089K02Rik	269.09	6.50E-05	1.54E-02	10.34
9930024M15Rik	121.85	5.47E-05	1.37E-02	10.27
B3galt2	77.72	1.82E-04	2.86E-02	9.65
Gm15567	65.00	3.58E-04	4.33E-02	9.38
Cd177	2444.16	2.49E-04	3.59E-02	9.15
Sirpb1a	176.23	6.17E-05	1.49E-02	8.98
Gm45762	219.93	7.80E-06	3.72E-03	8.61
Gm45534	88.90	1.59E-04	2.61E-02	7.13
Gm36933	1086.56	2.92E-13	5.55E-09	5.39
Ighv9-4	1181.42	1.19E-06	1.07E-03	-5.11
Ighg3	14544.97	4.69E-05	1.23E-02	-5.53
Igkv4-69	372.67	9.32E-05	1.86E-02	-5.55
Ighv1-26	906.16	5.17E-06	2.97E-03	-5.86
Ighv1-52	320.79	1.49E-04	2.56E-02	-7.34
Avil	33.89	3.96E-04	4.64E-02	-29.73
Gm48899	103.37	1.64E-05	5.90E-03	-88.25

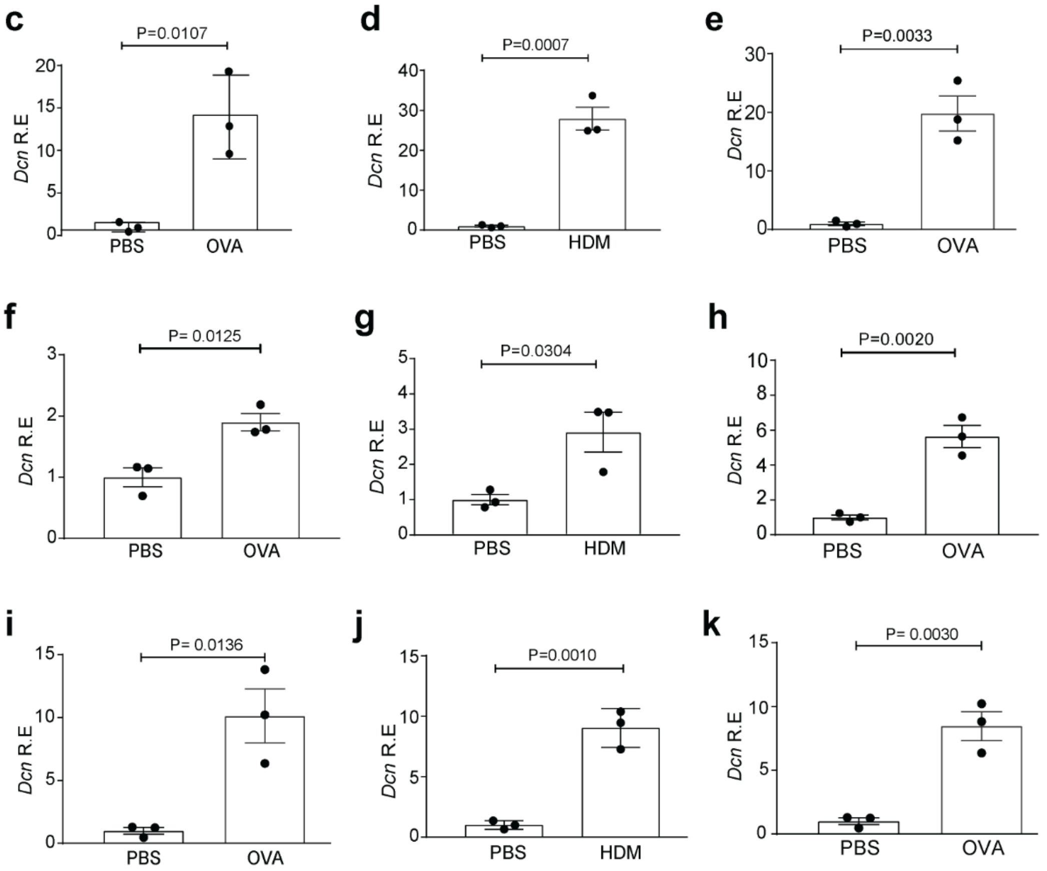

Supplementary Fig. 5. (a) PCA plot generated from PBS- and OVA-treated $CD3^+$ T cell RNA sequencing data (n=5). **(b)** List of differentially expressed genes, with log-fold changes ≥5 in T cells from OVA-, relative to PBS-treated, *Nf1*^{OPG} mice. All data are presented as the mean ± SEM. Increased *Dcn* RNA expression was detected in $CD3^+$ T cells from the spleen **(c-e)**, cervical lymph nodes **(f-h)** and optic nerve (**i-k**) of *Nf1*^{OPG} mice treated with OVA and HDM from 4 to 6 weeks of age and harvested at **(c, d, f, g, i, j)** 12 and **(e, h, k)** 24 weeks of age (n=3). A two-tailed Student's *t*-test was used. P values are indicated within each panel. From left to right in each panel: (**c**) P=0.0107; (**d**) P=0.0007; (**e**) P=0.0033; (**f**) P=0.0125; (**g**) P=0.0304, (**h**) P=0.0020; (**i**) P=0.0136; (**j**) P=0.0010; (**k**) P=0.0030.

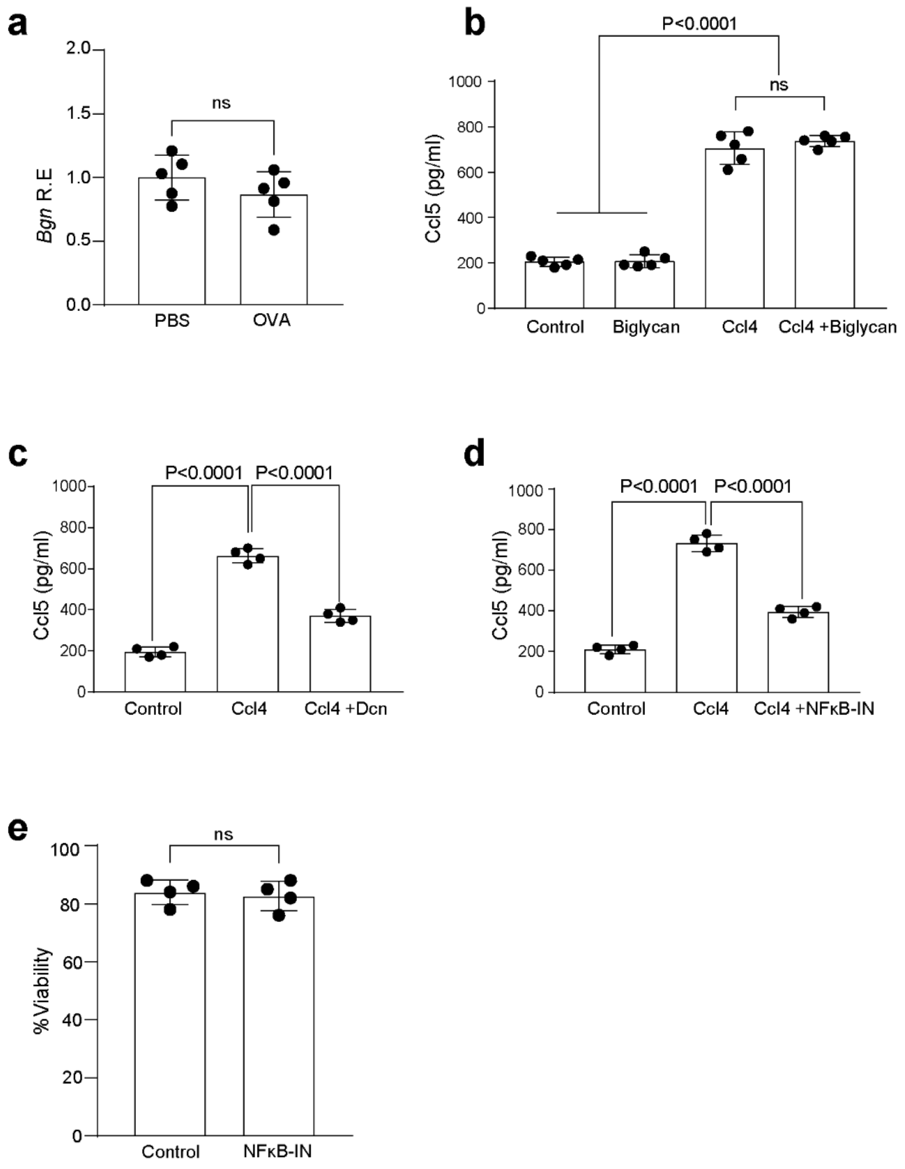

Supplementary Fig. 6. **(a)** OVA-treated *Nf1*^{OPG} optic nerves do not harbor increased biglycan (*Bgn*) RNA expression (*n*=5). (**b**) WT microglia were treated with either biglycan (25 µg/ml) alone or in combination with Ccl4 (6000pg/ml). Biglycan alone or in combination with Ccl4 does not induce microglia Ccl5 production (*n*=5). **(c)** Decorin inhibits increased microglia Ccl5 production in response to Ccl4 treatment *in vitro* (n=4). **(d)** NFkB inhibition (CAPE) blocks microglia Ccl5 production in response to Ccl4 treatment *in vitro* (n=4). (**e**) NFκB inhibition (CAPE) had no effect on microglia viability, as measured using a WST-1 cell viability assay (n=4). A one-way ANOVA with Bonferroni post-test correction was used. A two-tailed Student's *t*-test was used. **(a-e)** All data are presented as the mean ± SEM. From left to right in each panel: (**a**) ns; (**b**) P<0.0001, ns; (**c**) P<0.0001, P<0.0001; (**d**) P<0.0001, P<0.0001; (**e**) ns.

SUPPLEMENTARY TABLES

Supplementary Table 1. Antibodies used.

Antibody	Host	Source	Dilution
β-Actin (WB)	mouse	Cell Signaling, 58169S	1:1000
CD3 (IHC)	rat	Abcam, 11089	1:50
CD4 (IHC)	goat	R&D systems, AF554-SP	1:50
CD8α (IHC)	rabbit	Cell Signaling, 98941S	1:500
FOXP3 (IHC)	rabbit	Thermo scientific, 700914	1:100
HDAC1	mouse	Cell Signaling, 5356S	1:1000
Iba1 (IHC/IF)	rabbit	Wako, 019-19741	1:500
IκBα (WB)	rabbit	Cell Signaling, 4812S	1:1000
Phospho-IκBα (WB)	rabbit	Cell Signaling, 2859S	1:1000
Ki67 (IHC)	mouse	BD Pharmingen,550609	1:400
NFκB p65	rabbit	Cell Signaling, 8242S	1:1000
Phospho-NFκB p65	rabbit	Cell Signaling, 3033S	1:1000
TMEM119 (IF)	rabbit	Abcam, 209064	1:500

Supplementary Table 2. qRT-PCR probes used.

Gene	Probe set
Bgn (mouse)	Mm01191753_m1(TaqMan Gene Expression)
Ccl4 (mouse)	Mm00443111_m1(TaqMan Gene Expression)
Ccl5 (mouse)	Mm01302427_m1(TaqMan Gene Expression)
Dcn (mouse)	Mm00442039_m1(TaqMan Gene Expression)
Gapdh (mouse)	Mm99999915_g1 (TaqMan Gene Expression); internal control
Il1β (mouse)	Mm00434228_m1(TaqMan Gene Expression)
Il3 (mouse)	Mm00439631_m1(TaqMan Gene Expression)
Il4 (mouse)	Mm00445259_m1(TaqMan Gene Expression)
Il5 (mouse)	Mm00439646_m1(TaqMan Gene Expression)
Il6 (mouse)	Mm00446190_m1(TaqMan Gene Expression)
Il10 (mouse)	Mm01288386_m1(TaqMan Gene Expression)
Tnfα (mouse)	Mm00443258_m1(TaqMan Gene Expression)
