# Supplementary material for: Asthma reduces glioma formation by T cell decorin-mediated inhibition of microglia
Source: Nat Commun. 2021 Dec 8;12:7122. doi: 10.1038/s41467-021-27455-6 (PMC8654836; doi:10.1038/s41467-021-27455-6)
Supplement: Supplementary file 2 — Reporting Summary [file 41467_2021_27455_MOESM2_ESM.pdf]

nature portfolio

Corresponding author(s): David H. Gutmann, MD, PhD

Last updated by author(s): Nov 15, 2021

Reporting Summary

Nature Portfolio wishes to improve the reproducibility of the work that we publish. This form provides structure for consistency and transparency in reporting. For further information on Nature Portfolio policies, see our Editorial Policies and the Editorial Policy Checklist.

Statistics

For all statistical analyses, confirm that the following items are present in the figure legend, table legend, main text, or Methods section.

n/a	Confirmed	
☐	☒	The exact sample size (*n*) for each experimental group/condition, given as a discrete number and unit of measurement
☐	☒	A statement on whether measurements were taken from distinct samples or whether the same sample was measured repeatedly
☐	☒	The statistical test(s) used AND whether they are one- or two-sided<br>*Only common tests should be described solely by name; describe more complex techniques in the Methods section.*
☐	☒	A description of all covariates tested
☐	☒	A description of any assumptions or corrections, such as tests of normality and adjustment for multiple comparisons
☐	☒	A full description of the statistical parameters including central tendency (e.g. means) or other basic estimates (e.g. regression coefficient) AND variation (e.g. standard deviation) or associated estimates of uncertainty (e.g. confidence intervals)
☐	☒	For null hypothesis testing, the test statistic (e.g. *F*, *t*, *r*) with confidence intervals, effect sizes, degrees of freedom and *P* value noted<br>*Give P values as exact values whenever suitable.*
☒	☐	For Bayesian analysis, information on the choice of priors and Markov chain Monte Carlo settings
☒	☐	For hierarchical and complex designs, identification of the appropriate level for tests and full reporting of outcomes
☒	☐	Estimates of effect sizes (e.g. Cohen's *d*, Pearson's *r*), indicating how they were calculated

Our web collection on statistics for biologists contains articles on many of the points above.

Software and code

Policy information about availability of computer code

Data collection	Zeiss AxioScan-Z1, Image Studio Lite Version 5.2 software, Bio-Rad CFX Manager 3.0, LAS AF Lite 3.2.0 and Bio-Rad Imark Mpm6 6.2 were used to collect data.
Data analysis	GraphPad Prism 8.2.1 (GraphPad Software), ImageJ and Image Studio Lite Version 5.2 software were used for data analysis.

For manuscripts utilizing custom algorithms or software that are central to the research but not yet described in published literature, software must be made available to editors and reviewers. We strongly encourage code deposition in a community repository (e.g. GitHub). See the Nature Portfolio guidelines for submitting code & software for further information.

Data

Policy information about availability of data

All manuscripts must include a data availability statement. This statement should provide the following information, where applicable:
- Accession codes, unique identifiers, or web links for publicly available datasets
- A description of any restrictions on data availability
- For clinical datasets or third party data, please ensure that the statement adheres to our policy

Data availability. Supporting data are included in the Supplemental Materials section. Relevant GEO accession number are provided for all the datasets used and genrated.

Field-specific reporting

Please select the one below that is the best fit for your research. If you are not sure, read the appropriate sections before making your selection.

- [x] Life sciences
- [ ] Behavioural & social sciences
- [ ] Ecological, evolutionary & environmental sciences

For a reference copy of the document with all sections, see nature.com/documents/nr-reporting-summary-flat.pdf

Life sciences study design

All studies must disclose on these points even when the disclosure is negative.

Sample size	Sample sizes were chosen based on prior power calculations (80% confidence to detect 25% differences) and previously published experiments in our laboratory (Hegedus B, Cancer Research 2008; Kaul A, Neuro-Oncol 2015).
Data exclusions	No data were excluded
Replication	Multiple independently generated samples (the n is disclosed for each experiment at the Methods section) were used for all experiments. All attempts at replication were successful.
Randomization	Animals were randomly assigned to experimental groups.
Blinding	When possible, investigators were blinded to group allocation during data collection and analysis.

Reporting for specific materials, systems and methods

We require information from authors about some types of materials, experimental systems and methods used in many studies. Here, indicate whether each material, system or method listed is relevant to your study. If you are not sure if a list item applies to your research, read the appropriate section before selecting a response.

Materials & experimental systems

n/a	Involved in the study	
[ ]	[x]	Antibodies
[x]	[ ]	Eukaryotic cell lines
[x]	[ ]	Palaeontology and archaeology
[ ]	[x]	Animals and other organisms
[x]	[ ]	Human research participants
[ ]	[x]	Clinical data
[x]	[ ]	Dual use research of concern

Methods

n/a	Involved in the study	
[x]	[ ]	ChIP-seq
[x]	[ ]	Flow cytometry
[ ]	[x]	MRI-based neuroimaging

Antibodies

Antibodies used	Supplier names, catalog numbers, and working concentrations are provided in Supplemental Table 1.
Validation	Each antibody has been validated in the literature, company manuals, or was validated for the relevant species and tissue in our laboratory. We confirmed that each antibody achieved expected cellular localization and/or cell expression patterns by immunohistochemistry or expected sizes in immunoblotting.

Animals and other organisms

Policy information about studies involving animals; ARRIVE guidelines recommended for reporting animal research

Laboratory animals	All mouse strains were maintained on a strict C57BL/6 background, including wild type, Nf1 flox/mut; GFAP-Cre, and Nf1+/- mice. Both male and female mice were used at the ages specified in the manuscript. Mice were housed in barrier facilities with controlled light-dark cycles (12:12 hour) and ad libitum access to food and water.
Wild animals	No wild animals were used.
Field-collected samples	No field-collected samples were used.
Ethics oversight	All mice were used in accordance with an approved Animal Studies Committee protocol at Washington University.

Note that full information on the approval of the study protocol must also be provided in the manuscript.

Clinical data

Policy information about clinical studies

All manuscripts should comply with the ICMJE guidelines for publication of clinical research and a completed CONSORT checklist must be included with all submissions.

Clinical trial registration	*Provide the trial registration number from ClinicalTrials.gov or an equivalent agency.*
Study protocol	*Note where the full trial protocol can be accessed OR if not available, explain why.*
Data collection	*Describe the settings and locales of data collection, noting the time periods of recruitment and data collection.*
Outcomes	*Describe how you pre-defined primary and secondary outcome measures and how you assessed these measures.*

Magnetic resonance imaging

Experimental design

Design type	T1-weighted images with contrast enhancement using MnCl2 -- Manganese Enhanced Magnetic Resonance Imaging. (MEMRI).
Design specifications	In vivo detection and measurement of optic nerve in mouse models of NF1 (including wild type) with MEMRI.
Behavioral performance measures	n/a

Acquisition

Imaging type(s)	Anatomic structure via T1-weighted images.
Field strength	4.7 Tesla.
Sequence & imaging parameters	T1W image were collected with 2D Spin-Echo multiple slice sequence (SEMS): TR/TE, 300ms/11 ms; Matrix size, 128x128; FOV, 16 x 16 mm^2; Image slices, 15; Slice thickness, 0.5 mm; Averages, 16 (block size) x 2 =32 total scans (20 minutes).
Area of acquisition	The 6th slice (out of 15) was centered on the accessory olfactory bulbs of the mouse brain.

Diabetes MRI

Diffusion MRI ☐ Used ☒ Not used

Preprocessing

Preprocessing software	MATLAB (R2020a); ITK-SNAP (version, 3.8.0).
Normalization	Using Matlab, MR images were zero-padded and a Gaussian filter (Sigma 0.75) was applied, after which the data were converted to NIfTI format and exported. For each animal, a representative single slice from the T1-weighted images of the optic nerve was segmented and the optic-nerve cross-sectional area calculated with ITK-SNAP.
Normalization template	n/a
Noise and artifact removal	n/a
Volume censoring	n/a

Statistical modeling & inference

Model type and settings	n/a
Effect(s) tested	n/a

Specify type of analysis: ☐ Whole brain ☒ ROI-based ☐ Both

Anatomical location(s): optic nerves/chiasm

Statistic type for inference (See Eklund et al. 2016)	n/a
Correction	n/a

Models & analysis

n/a	Involved in the study
☒	☐ Functional and/or effective connectivity
☒	☐ Graph analysis
☒	☐ Multivariate modeling or predictive analysis
